# Effects of bacterial fertilizer and soil amendment on *Spuriopinella brachycarpa* (Kom.) Kitag. growth and soil microbiota

**DOI:** 10.3389/fmicb.2025.1560982

**Published:** 2025-04-25

**Authors:** Yue Zou, Yan Zou, Baiming Yang, Jianlei Qiao, Shuang Liu, Chunbo Zhao, Kun Shi, Yue Yu, Shuang Li, Shanshan Chen

**Affiliations:** ^1^College of Horticulture, Jilin Agricultural University, Changchun, China; ^2^Liaoyuan Sino-German Modern Agriculture Co., Ltd., Dongliao, China; ^3^Teaching and Research Base Management Office, Jilin Agricultural University, Changchun, China

**Keywords:** *Spuriopinella brachycarpa*, bacterial fertilizer, soil amendment, root architecture, soil fertility, microbial diversity

## Abstract

**Introduction:**

*Spuriopinella brachycarpa* (Kom.) Kitag. is a nutritious wild vegetable, but its quality deteriorates during artificial cultivation due to soil condition alterations. Microbial fertilizers and soil amendments hold promise for improving cultivation outcomes, yet their combined effects remain under - explored.

**Methods:**

A field experiment was conducted with seven treatments, including a control (CK) and six combinations of *Trichoderma harzianum*, *Bacillus subtilis*, and earthworm polysaccharide. Plant samples were analyzed for yield, quality, and root architecture, while soil samples were tested for fertility and microbial community characteristics.

**Results:**

Treatments T3 (dual bacterial fertilizers) and T6 (dual bacterial fertilizers + soil amendment) significantly enhanced yield, nutritional quality, and root development. T6 also maintained high soil fertility and optimized the soil microbial community in terms of richness, diversity, and beneficial species abundance.

**Discussion:**

The positive effects of T3 and T6 are likely due to the synergy between the bacterial fertilizers and the soil amendment, which improves nutrient cycling, soil structure, and microbial functions. However, the study has limitations, such as the need for long - term research and more in - depth exploration of microbial functions.

## Graphical Abstract

Action mechanism of soil improver and microbial fertilizer on plants and soil microorganisms. 
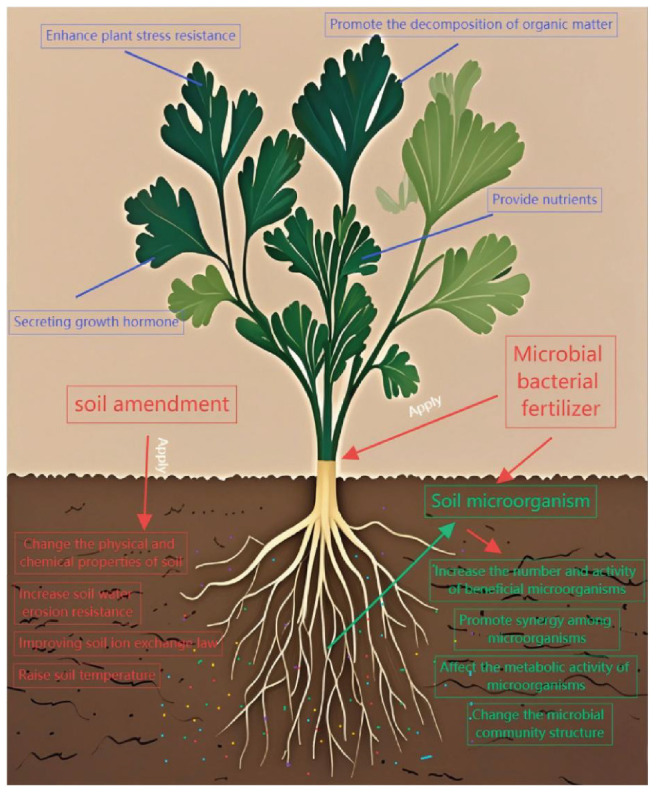

## Introduction

*Spuriopinella brachycarpa* (Kom.) Kitag., a perennial herb of the Umbelliferae family, thrives in cool, humid mountainous habitats, particularly in broad-leaved forests and valley wetlands (Yang et al., 2000). Its tender stems and leaves are prized as a wild vegetable due to their high nutritional value, including protein, vitamin C, and flavonoids (Li et al., 2016). However, under artificial cultivation, *Spuriopinella brachycarpa* (Kom.) Kitag. carpa often exhibits reduced quality and yield, primarily attributed to deviations in soil conditions from its natural rhizosphere environment—characterized by rich organic matter, optimal drainage, and a deep soil layer (Chen et al., 2024). Recent studies demonstrate that microbial fertilizers can mitigate salt stress in saline soils by reducing sodium accumulation and enhancing nutrient availability (Liu et al., 2023), providing a sustainable solution for improving rhizosphere health.

As shown in the mechanism diagram in Graphical abstract. Recent studies suggest that microbial fertilizers and soil amendments can mitigate cultivation challenges by enhancing nutrient cycling and modifying soil structure (Huang et al., 2019; Wu Y. et al., 2024). For instance, *Trichoderma harzianum* and *Bacillus subtilis* are known to solubilize phosphorus, suppress pathogens, and stimulate root growth (Zhang et al., 2023a), A recent meta-analysis by Mannai and Boughalleb-m’Hamdi (2023) demonstrated that combined application of these two microbes increased phosphorus availability by 40% compared to single-strain treatments through complementary enzymatic activities, with *T. harzianum* producing cellulases to degrade organic matter and *B. subtilis* secreting phosphatases to solubilize inorganic phosphorus. d’Errico et al. (2022) confirmed that Trichoderma harzianum combined with organic fertilizer can enhance the inhibition effect on nematodes and promote the growth and yield of tomatoes.

Soil amendments serve as critical regulators of soil functions, playing multi-dimensional roles in modern agriculture. Their core functions include: Physical improvement: By promoting the formation of water-stable aggregates, reducing soil bulk density, and enhancing porosity and water retention capacity. For example, wheat bran-chicken manure composite amendments can increase soil saturated hydraulic conductivity by 30% (Qi et al., 2015). Chemical regulation: Through adsorption of heavy metal ions, pH adjustment, and enhancement of cation exchange capacity to improve the rhizosphere microenvironment. Zeolite and humic acid-based materials, for instance, effectively passivate cadmium, arsenic, and other pollutants in soils (Zhang et al., 2023a). Biological activation: By providing organic carbon sources to promote beneficial microbial proliferation. Earthworm polysaccharides, for example, increase soil microbial biomass carbon by 25% and significantly enhance urease and phosphatase activities (Cruz - Méndez et al., 2021). Notably, polymer-based amendments (e.g., polyacrylamide) demonstrate remarkable efficacy in reducing soil erosion, achieving over 40% reduction in water and soil loss (Lee et al., 2010). These properties position soil amendments as key tools for constructing healthy rhizosphere microecology and achieving fertilizer reduction with efficiency gains.

Against the backdrop of increasing demand for sustainable agriculture, current studies have largely focused on individual microbial inoculants or soil amendments, leaving critical gaps in understanding their interactive effects. This study addresses this knowledge deficit by systematically investigating the synergistic integration of *Trichoderma harzianum* and *Bacillus subtilis* consortia with earthworm polysaccharide amendments. Through a replicated field trial, we aim to elucidate the combined effects on *Spuriopinella brachycarpa* yield and root architectural plasticity, assess dynamic changes in soil nutrient cycling and physical-chemical properties, and characterize rhizosphere microbial community shifts at taxonomic and functional levels. We hypothesize that this integrative approach will reconstruct a natural-like rhizosphere microenvironment through three complementary mechanisms: 1. microbial niche complementarity where *T. harzianum* enhances biocontrol efficacy while *B. subtilis* improves nutrient solubilization; 2. soil matrix modification via earthworm polysaccharide-stabilized aggregates; and 3. functional synergy optimizing carbon-nitrogen metabolism for sustained soil fertility and disease suppression. This research seeks to provide mechanistic insights for developing precision soil health management strategies that reconcile productivity and sustainability.

## 2 Materials and methods

### 2.1 Field experiment

In September 2023, sufficient plump seeds of *Spuriopinella brachycarpa* (Kom.) Kitag. were collected in Tonghua County, Tonghua City, Jilin Province. After being dried in the shade, the seeds were sown in the facility base of Jilin Agricultural University in October of the same year. After overwintering and emerging, the seedlings with a height of about 10 cm were transplanted in June 2024. The open-field ridge cultivation method was adopted, with a plant spacing of 5 cm × 10 cm. Each plot had an area of 1 m^2^ with three replicates and was randomly arranged. After planting the seedlings of mountain celery, a 60% shade net was immediately installed. When the soil moisture, monitored by a soil humidity detector, dropped below 60% of field capacity, watering was carried out manually using a sprinkler. Weeding was done manually every 2 weeks. The initial soil nutrient levels were determined by five-point sampling before fertilization. Soil properties included pH (6.2 ± 0.3), organic matter (2.8% ± 0.4), alkali-hydrolyzable nitrogen (128.33 mg/kg), Olsen P (35.25 mg/kg), and exchangeable K (135.22 mg/kg).

A total of seven treatments were set up in the experiment, with the conventional artificial cultivation of *Spuriopinella brachycarpa* (Kom.) Kitag. as the control (CK). The other treatments and corresponding numbers are shown in Table 1. The two bacterial fertilizers used in the experiment were produced by the British Bell Biotechnology Co., Ltd. The effective viable count of *Trichoderma harzianum* fertilizer was ≥ 10 billion/g, and that of *Bacillus subtilis* fertilizer was ≥ 40 billion/g. The soil amendment used was the earthworm polysaccharide produced by Tianjin Comet Biotechnology Co., Ltd. The fertilization amount was the recommended dosage by the manufacturer (both *Trichoderma harzianum* fertilizer and *Bacillus subtilis* fertilizer were diluted 1000 times with water at 1.5 g/m^2^ and applied with watering, and the earthworm polysaccharide was evenly spread at 30 g/m^2^), this dosage aligns with the international guidelines for Bacillus-based biofertilizers in vegetable cultivation (Luan et al., 2020). Each treatment was applied with 3,000 kg/667 m^2^ of organic fertilizer as the base fertilizer. The organic fertilizer used was commercially available cow manure compost (N-P_2_O_5_-K_2_O = 3-2-1, total organic matter ≥ 45%) produced by Jilin Agricultural University Organic Fertilizer Factory.

**TABLE 1:** Test treatment and corresponding numbers.

Treatment	Organic fertilizer	*Trichoderma harzianum*	*Bacillus subtilis*	Earthworm polysaccharide
CK	4.5 kg/m^2^			
T1	4.5 kg/m^2^	1.5 g/m^2^		
T2	4.5 kg/m^2^		1.5 g/m^2^	
T3	4.5 kg/m^2^	1.5 g/m^2^	1.5 g/m^2^	
T4	4.5 kg/m^2^	1.5 g/m^2^		30 g/m^2^
T5	4.5 kg/m^2^		1.5 g/m^2^	30 g/m^2^
T6	4.5 kg/m^2^	1.5 g/m^2^	1.5 g/m^2^	30 g/m^2^

### 2.2 Plant sampling and detection of yield and plant physicochemical properties

When *Spuriopinella brachycarpa* (Kom.) Kitag. grew to about 25 cm, it was harvested. The harvest time should be in the morning or evening, and the stubble should be left 1–2 cm at the base. At harvest, the yield was counted using a kilogram scale (yield per mu = plot yield × 667), and intact and disease-free *Spuriopinella brachycarpa* (Kom.) Kitag. plants were selected and brought back to the laboratory for determination of quality indicators, including soluble sugar, soluble protein, vitamin C (Vc), total flavonoids, and coumarin. The soluble protein content was determined by the Coomassie Brilliant Blue G—250 staining method at 595 nm, the Vc content was determined by the molybdenum blue colorimetric method at 760 nm, the soluble total sugar content was determined by the anthrone colorimetric method at 620 nm (Kong and Yi, 2008), and the total flavonoid content was determined by the colorimetric method (Yang and Yang, 2021).

### 2.3 Root sampling and determination of root system architecture

During the harvest period, the root system of *Spuriopinella brachycarpa* (Kom.) Kitag. was sampled by the stratified sampling method. Five plants with uniform growth were selected. With *Spuriopinella brachycarpa* (Kom.) Kitag. as the center, the length was twice the plant spacing and the width was the single row spacing. Each 10 cm was regarded as a soil layer unit, and the sampling depth was 50 cm. The root system in each soil layer mixture was taken out, washed with water, and all roots were picked out and scanned with a WinRHIZO LA 2400 scanner for root length and root surface area.

### 2.4 Soil sampling and determination of soil basic fertility indicators

Before fertilization, the original soil samples were collected, and on the 10th, 20th, 30th, 40th, 50th, and 60th days after planting, soil samples were collected from each plot by the five-point sampling method using a cutting ring. Each treatment was repeated three times. The soil samples were placed in a cool and ventilated place to dry naturally and then sieved for determination of soil basic fertility. The alkali-hydrolyzable nitrogen content was determined by the alkali-hydrolysis diffusion method, the Olsen P levels was determined by the molybdenum-antimony anti-colorimetric method, and the Exchangeable K levels was determined by the flame photometry method (Bao, 2000).

### 2.5 Soil microorganism detection

Soil sampling: During the harvest of *Spuriopinella brachycarpa* (Kom.) Kitag., the soil at the root of *Spuriopinella brachycarpa* (Kom.) Kitag. was collected by the root shaking method. Each treatment was repeated three times. The collected soil was passed through liquid nitrogen and stored in a –80°C refrigerator.

Soil microorganism DNA extraction: The total DNA of the microbial community was extracted from the collected soil samples by the CTAB method (Schenk et al., 2023). The quality of DNA extraction was detected by agarose gel electrophoresis, and the DNA was quantified by an ultraviolet spectrophotometer.

#### 2.5.1 PCR amplification

The primers for bacterial amplification were 341F (5′-CCTACGGGNGGCWGCAG-3′) and 805R (5′-GACTACHVGGGTATCTAATCC-3′), and the V3–V4 variable region of the bacterial 16S rDNA gene was amplified by PCR. The primers were designed and synthesized by Hangzhou Lianchuan Biotechnology Co., Ltd. Ultra-pure water was used throughout the DNA extraction process to exclude the possibility of false positive PCR results as a negative control. The PCR reaction system was 25 μL: 12.5 μL Phusion Hot start flex 2X Master Mix, 2.5 μL Forward Primer, 2.5 μL Reverse Primer, 50 ng DNA, and ddH2O was added to 25 μL. The reaction parameters were: pre-denaturation at 98°C for 5 min; denaturation at 98°C for 10 s, annealing at 54°C for 30 s, and extension at 72°C for 45 s for 35 cycles; and final extension at 72°C for 10 min. The PCR amplification products were detected by 2% agarose gel electrophoresis, and the PCR products were purified by AMPure XT beads (Beckman Coulter Genomics, Danvers, MA, United States) and quantified by Qubit (Invitrogen, United States) (Kao et al., 2009; Pecundo et al., 2021).

#### 2.5.2 Data analysis samples

The paired-end data obtained by high-throughput sequencing were spliced and filtered. The primer sequences and balanced base sequences of the RawData were removed using the cutadapt (v1.9) software. Each pair of paired-end reads was spliced and combined into a longer tag using the FLASH (v1.2.8) software according to the overlap region. The window was set to 100 bp by default during splicing, and the sequences shorter than 100 bp were removed after splicing. The chimera sequences were removed using the Vsearch (v2.3.4) software. The OTU clustering analysis was performed using R—3.4.4 (VennDiagram), and the single-sample composition analysis, including Chao, Shannon, Simpson indexes, etc., was performed using R—3.4.4 (ggplot). The species composition bar chart analysis was performed using R—3.4.4 (ggplot2) based on the relative abundance of species. The RDA analysis was performed using R—3.4.4 (vegan).

Data analysis was performed using SPSS Statistics 26 software, ANOVA by univariateDuncan, and significance at the *p* < 0.05 level.

## 3 Results and analysis

### 3.1 Effects of different bacterial fertilizer and soil amendment treatments on the quality and yield of *Spuriopinella brachycarpa* (Kom.) Kitag.

As shown in Table 2, after the application of bacterial fertilizer and soil amendment, the contents of soluble sugar, soluble protein, Vc, total flavonoids, and yield were increased to varying degrees. T3 and T6 significantly increased soluble sugar (11.96 mg/g and 12.2 mg/g) compared to other treatments (*p* < 0.05). T1 and T2 had higher soluble sugar (10.65–10.82 mg/g) than CK (9.41 mg/g). T6 had the highest soluble protein (13.54 mg/g), followed by T5 (13.13 mg/g) and T3 (12.56 mg/g), all significantly higher than CK (10.23 mg/g) (*p* < 0.05). T3 had the highest vitamin C (92.87 mg/g), with T2 (90.19 mg/g) and T6 (87.12 mg/g) also higher than CK (79.51 mg/g) (*p* < 0.05). T6 had the highest total flavonoids (17.57 mg/g), followed by T3 (16.87 mg/g) and T5 (15.02 mg/g), all significantly higher than CK (12.04 mg/g) (*p* < 0.05). T3 and T6 had the highest yields (1365.67 kg/667 m^2^ and 1374.33 kg/667 m^2^), significantly higher than other treatments (*p* < 0.05). T2 and T5 also outperformed CK (1048.67 kg/667 m^2^).

**TABLE 2:** Effects of different bacterial fertilizers and soil amendments on the quality and yield of *Spuriopinella brachycarpa* (Kom.) Kitag.

	Soluble sugar content (mg/g)	Soluble protein (mg/g)	Vitamin C (mg/g)	Total flavonoids content (mg/g)	Yield (kg/667 m^2^)
CK	9.41 ± 0.42 d	10.23 ± 0.18 f	79.51 ± 2.77 de	12.04 ± 0.31 f	1048.67 ± 100.05 c
T1	10.65 ± 0.44 c	10.84 ± 0.42 ef	78.88 ± 3.89 e	11.88 ± 0.33 f	1060.00 ± 26.23 c
T2	10.82 ± 0.23 c	11.84 ± 0.94 cd	90.19 ± 2.32 ab	13.16 ± 0.39 e	1220.33 ± 37.90 b
T3	11.96 ± 0.20 a	12.56 ± 0.88 bc	92.87 ± 1.21 a	16.87 ± 0.59 b	1365.67 ± 51.87 a
T4	11.08 ± 0.22 bc	11.40 ± 0.08 de	81.77 ± 3.37 de	13.96 ± 0.28 d	1095.00 ± 34.89 c
T5	11.68 ± 0.41 ab	13.13 ± 0.28 ab	84.54 ± 3.08 cd	15.02 ± 0.23 c	1306.33 ± 91.63 ab
T6	12.2 ± 0.15 a	13.54 ± 0.24 a	87.12 ± 0.97 bc	17.57 ± 0.08 a	1374.33 ± 33.49 a

Data in this table are presented as mean ± standard deviation. Different letters in the same column indicate significant differences (*p* < 0.05) determined by one-way ANOVA followed by Duncan’s multiple range test.

### 3.2 Effects of different bacterial fertilizer and soil amendment treatments on the root system architecture of *Spuriopinella brachycarpa* (Kom.) Kitag.

**FIGURE 1:**
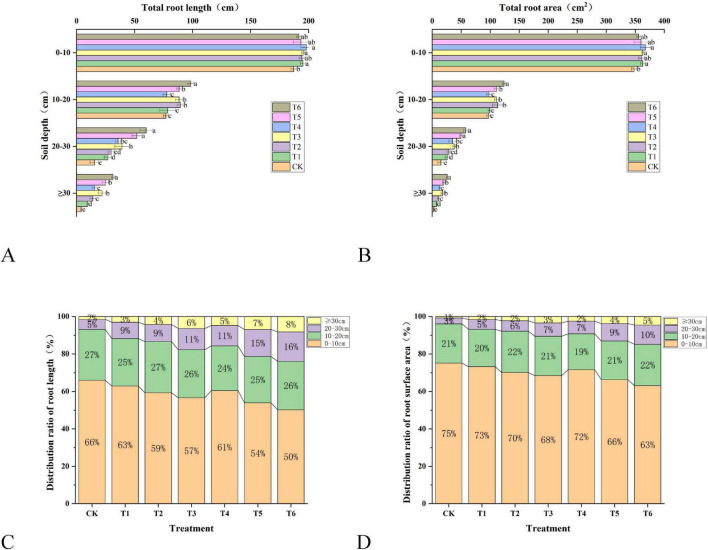
Effects of different bacterial fertilizers and soil amendments on the root configuration of *Spuriopinella brachycarpa* (Kom.) Kitag. **(A)** The total length of roots of *Spuriopinella brachycarpa* (Kom.) Kitag. in different soil depths under different treatments, **(B)** the total surface area of roots of celery in different soil depths under different treatments, **(C)** the proportion of roots of *Spuriopinella brachycarpa* (Kom.) Kitag. in different soil depths under different treatments, and **(D)** the percentage of roots in different soil depths under different treatments.

Figure 1 presents the effects of different bacterial fertilizers and soil amendments on the root configuration of *Spuriopinella brachycarpa* (Kom.) Kitag. Figure 1A shows the total root length at varying soil depths (0–10 cm, 10–20 cm, 20–30 cm, > 30 cm), with T6 exhibiting the most extensive root growth across all depths, indicating enhanced soil exploration. Figure 1B illustrates the total root surface area, which is largest in T6 at all depths, suggesting improved nutrient absorption capacity. Figure 1C displays the proportion of roots at different depths, where T6, T3, and T5 show a more balanced root distribution compared to CK, which has shallower root concentration. Panel D provides the percentage distribution of roots, highlighting that T6, T3, and T5 have a higher percentage of roots penetrating deeper layers (> 20 cm), enhancing nutrient and water uptake. Overall, T6 shows the most pronounced improvements in root architecture, indicating that the combined application of *Trichoderma harzianum*, *Bacillus subtilis* fertilizers, and earthworm polysaccharide creates a more favorable environment for root growth and development, ultimately improving plant performance and nutrient acquisition.

### 3.3 Effects of different bacterial fertilizer and soil amendment treatments on soil fertility

**FIGURE 2:**
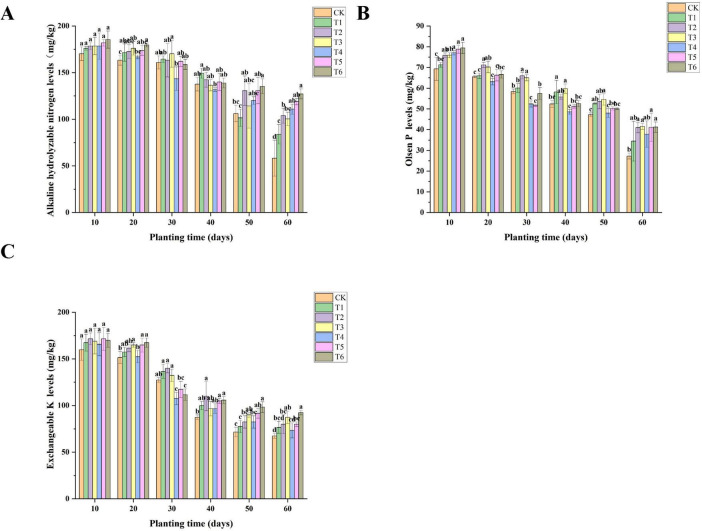
Effects of different bacterial fertilizers and soil amendments on soil fertility of *Spuriopinella brachycarpa* (Kom.) Kitag. root system. **(A)** The content of alkali-hydrolyzable nitrogen in soil under different treatments, **(B)** the levels of Olsen P in soil under different treatments, and **(C)** the levels of Exchangeable K in soil under different treatments.

As shown in Figure 2, the alkali-hydrolyzable nitrogen, Exchangeable K, and Olsen P decreased with the increase of cultivation time, and the three indicators were the highest at 10 days, and there was no significant difference between each treatment. From the data of alkali-hydrolyzable nitrogen, the control group CK showed an obvious downward trend within 60 days, decreasing from 170.333 to 58.333 mg/kg, which may be related to the gradual consumption of organic fertilizer. In contrast, the alkali-hydrolyzable nitrogen contents of the T1–T6 treatment groups were generally higher than that of CK at each time point, especially the T6 treatment, which still maintained a high level of 127.167 mg/kg at 60 days, indicating that the application of bacterial fertilizer and soil amendment helped maintain the nitrogen content in the soil. The data of Olsen P also showed a similar trend. The CK group decreased with time, while the T1–T6 treatment groups maintained a higher level. Especially, the Olsen P levels of the T6 treatment at 60 days reached 41.232 mg/kg, much higher than that of CK (27.248), indicating that the bacterial fertilizer and soil amendment had a significant effect on increasing the soil phosphorus content. The change of Exchangeable K was more complex. The CK group maintained a high level in the first 30 days and then decreased rapidly, while the T1–T6 treatment groups performed differently at different time points. Overall, the Exchangeable K levels of the T6 treatment was the highest at 60 days, reaching 92.454 mg/kg, which may be related to the comprehensive effect of the multiple bacterial fertilizers and soil amendment in the T6 treatment.

### 3.4 Effects of different bacterial fertilizer and soil amendment treatments on the root system soil microorganisms

#### 3.4.1 Alpha diversity analysis

From the observed_species, chao1, and ace indices in Table 3, the richness of the T6 treatment was the highest, followed by the T3 and T2 treatments, and the richness of the CK treatment was the lowest. From the Shannon index, the diversity of the T6 treatment was the highest, followed by the T3 and T2 treatments, and the diversity of the CK treatment was the lowest. From the Pielou index, the evenness of the T2, T6, and T3 treatments was relatively high, and the evenness of the CK treatment was the lowest. The Goods_coverage indices of all treatments were 1.00, indicating that the probability of new species not being detected in the sample was very low, and the sequencing result could well represent the true situation of the sample. In summary, under different treatments, the rhizosphere soil microorganisms of *Spuriopinella brachycarpa* (Kom.) Kitag. had the best performance in terms of richness, diversity, and evenness in the T6 treatment, while the CK treatment had the worst performance in these aspects. The coverage of all treatments was high, indicating that the representativeness of the sequencing result was good.

**TABLE 3:** Analysis of alpha diversity of soil microorganisms in the root system of *Spuriopinella brachycarpa* (Kom.) Kitag. with different bacterial fertilizers and soil amendments (alpha diversity indices were calculated using R software to evaluate the species richness and diversity of the soil microbial community.

	Observed_species	Shannon	Simpson	Chao1	Goods_coverage	Pielou_e	Ace
CK	2027.50	9.78	1.00	2028.27	1.00	0.89	2029.78
T1	2837.00	10.34	1.00	2843.60	1.00	0.90	2849.64
T2	3009.50	10.63	1.00	3016.14	1.00	0.92	3020.19
T3	3288.50	10.60	1.00	3293.94	1.00	0.91	3299.58
T4	2866.50	10.41	1.00	2869.53	1.00	0.91	2875.32
T5	3122.50	10.55	1.00	3125.75	1.00	0.91	3131.91
T6	3462.00	10.84	1.00	3465.14	1.00	0.92	3468.70

The Chao1, observed_species, and ace indices estimate the number of species in the community, while the Shannon and Simpson indices reflect the community diversity.

#### 3.4.2 BETA diversity analysis

**FIGURE 3:**
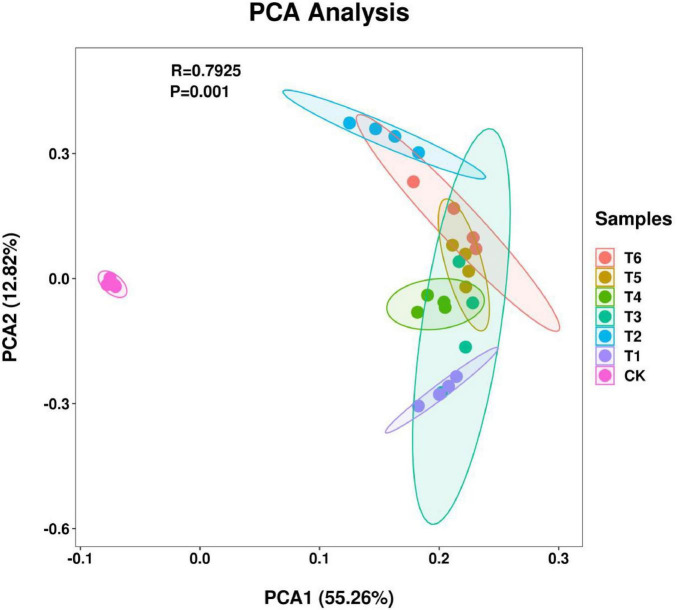
PCA diagram of rhizosphere microbial community distribution of *Spuriopinella brachycarpa* (Kom.) Kitag. Different colors in the graph represent different groups, points of the same color represent different samples in a group, and the same group is displayed in the form of a circle according to the 95% confidence interval. The closer the distance between the two samples, the more similar the microbial composition and structure between the samples, and the smaller the difference; On the contrary, it shows that the greater the difference of microbial composition and structure between samples.

As shown in Figure 3 (PCA plot), we can observe the differences in the microbial community structure of different treatment groups. The control group (CK) was clearly separated from the other treatment groups on PCA1, indicating that the application of bacterial fertilizer and soil amendment had a significant impact on the microbial community structure. The T1–T6 groups also showed certain separations on PCA1 and PCA2. Among them, the T1 and T2 treatments were distributed in the upper and lower halves of PAC2 and were far apart in the graph, indicating that there were significant differences in the microbial composition between the two, which may be caused by different bacterial fertilizer treatments. The T3, T4, T5, and T6 treatments were relatively concentrated because these four treatments had the same treatments in the bacterial fertilizer or soil amendment treatment groups. For example, the T3, T4, and T6 treatments were all applied with *Trichoderma harzianum* fertilizer, the T3, T5, and T6 were all applied with *Bacillus subtilis* fertilizer, and the T4, T5, and T6 were all applied with earthworm polysaccharide. The results showed that the bacterial fertilizer and soil amendment significantly changed the root microbiota environment of *Spuriopinella brachycarpa* (Kom.) Kitag., achieving the expected experimental results.

#### 3.4.3 Species composition analysis

**FIGURE 4:**
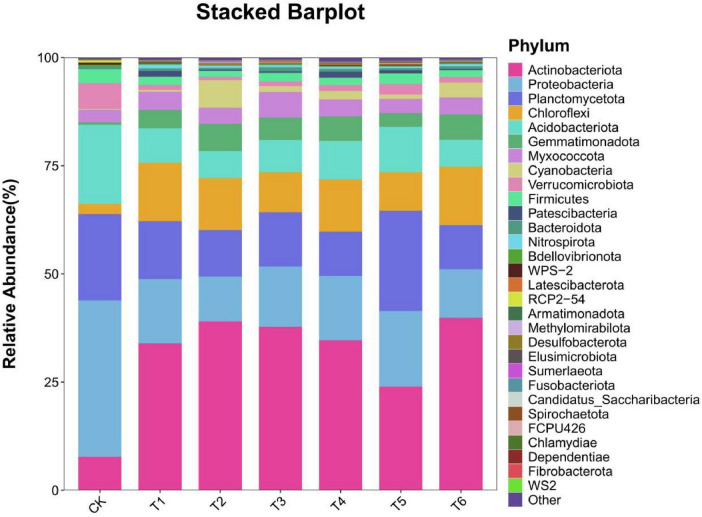
Composition analysis of soil microorganisms in the root system of *Spuriopinella brachycarpa* (Kom.) Kitag. under different treatments at the phylum. Select TOP30 species classification of abundance, and display the relative abundance of each sample/group in different forms. In the figure, the horizontal axis represents the sample name/group, and the vertical axis represents the relative abundance of a certain classification. Different colors correspond to different species at the same level. Through the histogram, we can understand the composition of highly expressed species in each sample/group, and also observe the composition and expression of species within the group and the trend of species expression between groups.

The stacked bar chart in Figure 4 shows the relative abundance percentages of different bacterial phyla under different treatments. The horizontal axis in the figure represents different treatment groups, and the vertical axis represents the percentage of relative abundance. The bar of each treatment group is divided into multiple color segments, and each color segment represents the relative abundance of a specific bacterial phylum. Through this figure, we can intuitively compare the differences in the relative abundances of various bacterial phyla in different treatment groups. As shown in Figure 4, in the CK treatment, the relatively abundant bacterial phyla were *Actinobacteriota*, *Proteobacteria*, and *Bacteroidota*. These bacterial phyla also occupied dominant positions in the T1–T6 treatments, which may be because all treatment groups used organic fertilizer as the base fertilizer, so they shared a similar basic microbial community structure to some extent. Compared with the CK treatment, the proportions of *Actinobacteriota*, *Chloroflexi*, and *Gemmatimonadota* in the T1–T6 treatments increased to varying degrees. The *proportions* of *Proteobacteria* and *Acidobacteriota* all showed a downward trend, indicating that different treatment measures had a significant impact on the soil microbial community structure at the phylum level. T6 treatment significantly increased the relative abundance of Actinobacteria (25.3% vs. CK 15.8%) and Gemmatimonadota (8.7% vs. CK 3.2%), which are known for organic matter decomposition and phosphorus solubilization (Geng et al., 2023). Conversely, the proportion of *Acidobacteria* decreased from 12.1% (CK) to 6.5% (T6), potentially due to competition suppression by introduced microbial consortia.

**FIGURE 5:**
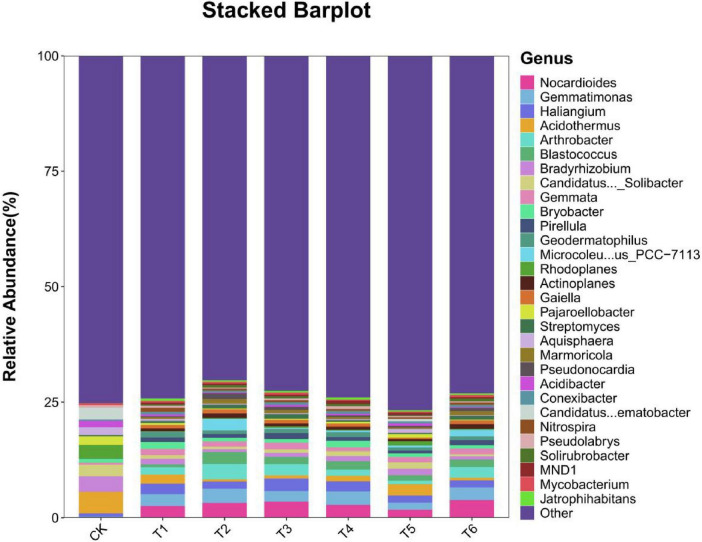
Composition analysis of soil microorganisms in the root system of *Spuriopinella brachycarpa* (Kom.) Kitag. under different treatments at the genus. The middle horizontal is the sample name/group, and the vertical axis represents the relative abundance of a classification; different colors correspond to different species at the same level. The bar chart can show composition of each sample/group with the high expression species and also observe the species!composition, expression, and expression trends between groups.

The stacked bar chart in Figure 5 shows the changes in the relative abundances of the soil microbial community structure at the genus level under different treatments. It can be seen that the relative abundances of various microbial genera were different under different treatments, reflecting the impact of treatment measures on the soil microbial community composition. In the CK treatment, each microbial genus showed a basic relative abundance distribution state, providing a benchmark for subsequent comparisons between treatments. For example, some common genera such as *Nocardioides* and *Arthrobacter* may have a certain relative abundance proportion in the control, representing the natural state of the soil microbial community without specific treatment. Compared with CK, the T1–T6 treatments had significant differences in the microbial flora structure, which may be because the bacterial fertilizer and soil amendment changed the soil environment and thus affected the microbial community structure. The dominant genera in the CK treatment were *Acidothermus*, *Bradyrhizobium*, *Candidatus_Solibacter*, and *Blastococcus*. The dominant genera in the T1–T6 treatments were *Nocardioides*, *Gemmatimonas*, *Haliangium*, and *Arthrobacter*.

#### 3.4.4 Environmental shadow analysis

**FIGURE 6:**
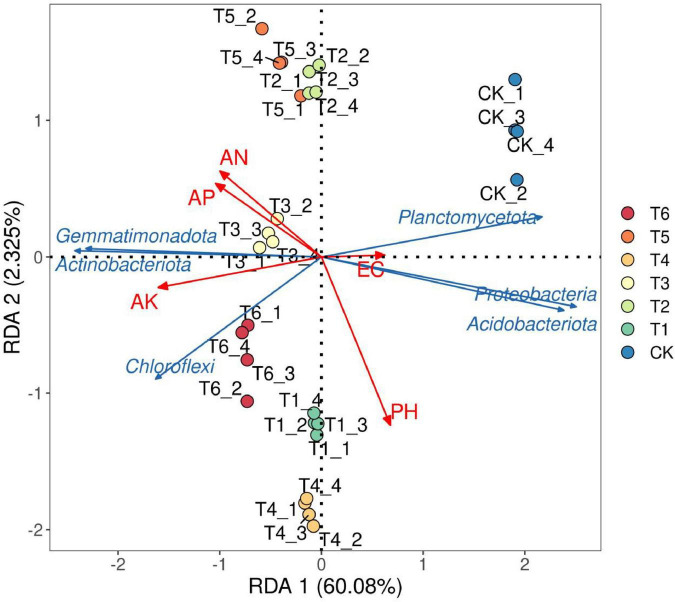
Distribution of microbial community in rhizosphere soil of *Spuriopinella brachycarpa* (Kom.) Kitag. and RDA analysis of environmental factors. In the red arrow in the legend, PH stands for pH, EC stands for conductivity, AK stands for Exchangeable K, AP stands for Olsen P, and AN stands for alkali-hydrolyzable nitrogen.

Redundancy analysis (RDA) is a PCA analysis of environmental factor constraints, reflecting the relationship between sample distribution and environmental factors. As can be seen from Figure 6, the sample points of the CK group were relatively concentrated, indicating that under the conventional artificial cultivation condition (only with organic fertilizer), the microbial community structure was relatively stable and similar. The sample points of each treatment group showed different characteristics. For example, after adding *Trichoderma harzianum* fertilizer in the T1 group, the sample points were significantly shifted relative to the CK group, which means that *Trichoderma harzianum* fertilizer significantly changed the microbial community structure. The sample point distribution of the T2 group using *Bacillus subtilis* fertilizer was unique, indicating that this bacterial fertilizer had a unique impact on the community structure. The sample point distributions of the T3, T4, T5, and T6 groups comprehensively reflected the changes in the microbial community structure under the combined action of multiple treatment factors due to different combinations of bacterial fertilizer and soil amendment. The directions and lengths of the arrows of the environmental factors (pH, EC, AP, AK, AN) in the figure reflected their influence degrees. The angle between the environmental factors AN and AP was acute, indicating a positive correlation between them. The angle between the environmental factor pH and other environmental factors (such as AN, AP, EC) was obtuse, indicating a negative correlation between them. The angle between the species *Gemmimonadota* and *Actinobacteriota* was acute, indicating a positive correlation between them. The angle between the species *Chloroflexi* and other species (such as *Gemmimonadota, Actinobacteriota*) was obtuse, indicating a negative correlation between them. The longer the arrow, the greater the impact of the factor on the sample treatment. For example, the arrows of the environmental factors AN and AP were longer, indicating that they were more affected by the sample treatment. The arrows of the species *Planctomycetota* and *Proteobacteria* were longer, indicating that they were more affected by the sample treatment. Taking pH as an example, its arrow pointed to the lower left, indicating that the soil pH had an important impact on the microbial community structure, and the sample point distribution in the lower left region may be more affected by the low pH value. The EC arrow pointed to the right, and the longer arrow indicated that the electrical conductivity (EC) had a significant impact on the community structure, and the sample point distribution on the right was closely related to EC. The directions and positions of the arrows of AP and AK also showed that they played an important role in shaping the microbial community structure.

#### 3.4.5 Microbial function prediction

**FIGURE 7:**
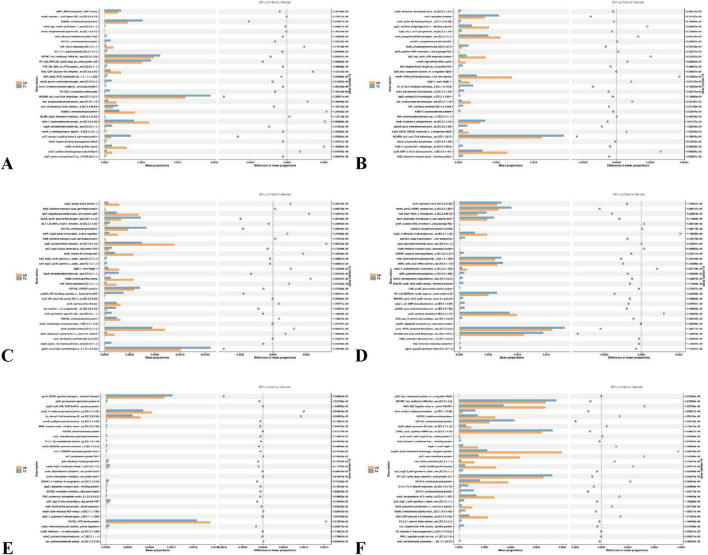
This Figure depicts the functional prediction of rhizosphere soil microbial communities based on the KEGG Orthology (KO) database (Release 106.0), generated using the Lianchuan Cloud Platform (Lyu et al., 2023). The heatmap illustrates differential functional gene abundances between six treatment groups (T1–T6) and the control (CK), with each subplot **(A–F)** representing pairwise comparisons: **(A)** (T1 vs. CK), **(B)** (T2 vs. CK), **(C)** (T3 vs. CK), **(D)** (T4 vs. CK), **(E)** (T5 vs. CK), and **(F)** (T6 vs. CK). Color intensity corresponds to Z-score normalized relative abundance (orange upregulated, blue downregulated), highlighting functional categories such as energy metabolism (e.g., acyl-CoA dehydrogenase in T1), amino acid biosynthesis (e.g., lysine metabolism in T6), stress response (e.g., outer membrane protein omp31 in T6), and nutrient cycling (e.g., iron carrier biosynthesis in T3). Notably, T6 uniquely upregulated lysY (lysine metabolism) and omp31 (oxidative stress resistance), while T3 showed enhanced gabD (γ-aminobutyric acid metabolism) and iucC (siderophore synthesis). Functional annotations were derived using DESeq2 (adjusted *p* < 0.05) and hierarchical clustering based on Bray-Curtis dissimilarity.

As can be seen from Figure 7 the functional prediction analysis of soil microbial communities in each treatment group compared to the control group (CK) revealed the following: T1 Treatment: Significantly enriched ACADM (acyl-CoA dehydrogenase) and BLMH (bleomycin hydrolase) functions. ACADM enhances fatty acid β-oxidation metabolism and promotes soil organic matter decomposition. BLMH is involved in antibiotic degradation and affects microbial competitive interactions. T2 Treatment: In addition to ACADM function enrichment, comGC (competence protein ComGC) function was significantly enhanced, increasing bacterial genetic transformation ability and promoting microbial genetic diversity. T3 Treatment: gabD (succinate-semialdehyde dehydrogenase) and iucC (iron carrier biosynthesis enzyme) functions were prominent. gabD enhances microbial stress resistance, and iucC optimizes iron nutrition cycling. T4 Treatment: purL (phosphoribosylglycinamide synthetase) and cysK (cysteine synthase A) functions were enriched. purL promotes microbial nucleic acid synthesis, and cysK regulates microbial proliferation and metabolic balance through sulfur metabolism. T5 Treatment: K07001 (uncharacterized protein) and frc (formyl-CoA transferase) functions showed significant differences, suggesting regulation of unknown metabolic pathways and formate metabolism. T6 treatment significantly enriched the omp31 gene (outer membrane immunogenic protein), which enhances microbial resistance to oxidative stress and pathogenic invasion (Kour et al., 2024). This functional improvement may be linked to autophagy induction, as autophagosomes sequester damaged proteins and organelles for degradation, thereby maintaining cellular homeostasis under stress (Klionsky et al., 2021). This functional improvement likely contributes to the stable soil nitrogen levels observed in T6 (Figure 2A), as stress-resistant microbes maintain nitrogen cycling efficiency even under environmental perturbations. At the same time, the enrichment of lysY (lysine metabolism-related enzyme) further optimizes the nitrogen metabolism pathway and promotes the conversion of organic nitrogen to ammonium nitrogen, thereby increasing the availability of soil nitrogen (He et al., 2012).

## 4 Discussion

The root system is the link between plants and the soil, and plants absorb nutrients from the soil through the root system for their growth (Freschet et al., 2021). In this study, by setting different treatments of bacterial fertilizer and soil amendment, the effects on the growth and development of *Spuriopinella brachycarpa* (Kom.) Kitag. and the diversity of the rhizosphere soil bacterial community were deeply explored. The results showed that these treatment measures had significant effects on the quality, yield, root architecture, soil fertility, and rhizosphere soil microbial community of *Spuriopinella brachycarpa* (Kom.) Kitag.

From the perspective of the growth and development of *Spuriopinella brachycarpa* (Kom.) Kitag., the quality indicators (such as soluble sugar, soluble protein, Vc, total flavonoids content) and yield were all improved to different degrees under different treatments. Among them, treatments T3 and T6 performed particularly well in terms of quality and yield, which may be attributed to the synergistic effect of *Trichoderma harzianum* fertilizer and *Bacillus subtilis* fertilizer. *Trichoderma harzianum* fertilizer and *Bacillus subtilis* fertilizer secrete various beneficial metabolites such as enzymes, antibiotics, and plant growth regulators in the soil (Azeem et al., 2020; Zhang et al., 2023b), which promote the absorption and utilization of nutrients by *Spuriopinella brachycarpa* (Kom.) Kitag. and enhance its stress resistance, thereby increasing the yield and quality. At the same time, the treatments with the combined application of soil amendment (T4, T5, T6) had more significant effects, indicating that the soil amendment, by adjusting the physical and chemical properties of the soil (Wu Q. et al., 2024), this is consistent with Ashwood and Frank’s research results on Alnus cordata through composting green waste (CGW). Ashwood et al. (2018) created a more favorable environment for the growth of *Spuriopinella brachycarpa* (Kom.) Kitag. and further enhanced the effect of the bacterial fertilizer. For example, earthworm polysaccharide may improve the soil aggregate structure, increase soil aeration and water retention, which is beneficial to root growth and nutrient absorption, and thus improve the growth performance of *Spuriopinella brachycarpa* (Kom.) Kitag.

In terms of root architecture, treatment T6 significantly enhanced root activity, enabling the roots to penetrate deeper into the soil to absorb nutrients. This may be due to the combined effect of the bacterial fertilizer and soil amendment in improving the soil environment and promoting root growth and development (Yan et al., 2024). *Trichoderma harzianum* fertilizer and *Bacillus subtilis* fertilizer may establish a symbiotic relationship with the roots, enhancing the ability of the roots to acquire nutrients and water (Li et al., 2024). At the same time, earthworm polysaccharide may improve the rhizosphere environment, stimulating root growth and branching, thus forming a more developed root architecture (Cruz - Méndez et al., 2021; Neuhoff et al., 2024). This change in root architecture helps *Spuriopinella brachycarpa* (Kom.) Kitag. better adapt to environmental changes during growth and improve the utilization efficiency of nutrients and water, providing more sufficient support for plant growth.

The application of bacterial fertilizer and soil amendment had a positive effect on maintaining the contents of alkali-hydrolyzable nitrogen, Olsen P, and Exchangeable K in the soil. Treatment T6 could still maintain relatively high contents of nitrogen, phosphorus, and potassium at 60 days, indicating that the comprehensive effect of multiple bacterial fertilizers and soil amendments was helpful for maintaining and improving soil fertility, which was consistent with the research results of Haider (2021). The microorganisms in the bacterial fertilizer participate in the transformation and cycling of nutrients in the soil, such as nitrogen fixation, phosphorus and potassium solubilization (Zheng et al., 2009; Wani et al., 2015), making the nutrients in the soil more easily absorbed and utilized by plants. At the same time, the soil amendment may reduce nutrient loss and improve the availability and stability of soil nutrients by regulating soil pH and improving soil structure (Ma, 2020).

In the rhizosphere soil microbial community, the Alpha diversity analysis showed that treatment T6 had the best performance in terms of richness, diversity, and evenness. This indicates that this treatment created a more suitable living environment for soil microorganisms, promoting the increase in the types and numbers of microorganisms. The addition of bacterial fertilizer and soil amendment may provide more nutrients and living space for microorganisms, changing the composition and structure of the soil microbial community, which was consistent with the research results of Wu Q. et al. (2024) and others. The BETA diversity analysis further confirmed the significant impact of the treatment measures on the microbial community structure, and there were obvious differences in the microbial compositions between different treatment groups. In the species composition analysis, the changes in the relative abundances of some bacterial phyla at the phylum level under different treatments reflected the directional regulation effect of the treatment measures on the soil microbial community. For example, the increase in the proportion of beneficial bacterial phyla such as *Actinobacteriota* may contribute to the decomposition of soil organic matter and nutrient cycling (Geng et al., 2023), thus having a positive impact on the growth of *Spuriopinella brachycarpa* (Kom.) Kitag. The changes in the dominant genera at the genus level also indicated that the bacterial fertilizer and soil amendment changed the competitive pattern of the microbial community, promoting the growth of beneficial microbial genera such as *Nocardioides*, *Gemmatimonas*, *Haliangium*, and *Arthrobacter*. The enrichment of *Nocardioides* may promote organic matter decomposition by secreting cellulase (Azeem et al., 2020), while the phosphorus-solubilizing function of *Gemmatimonas* (Zhang et al., 2023b) is directly related to the increase in soil available phosphorus content in group T6 (41.2 mg/kg). This functional synergy effect may be the core mechanism for the significant treatment effect of T6. We further propose that the synergistic effects of T3 and T6 treatments may stem from the interaction between *T. harzianum* and *B. subtilis*, which jointly enhance soil organic matter decomposition and nutrient solubilization. This is supported by the increased abundance of *Actinobacteria* (associated with organic matter degradation) and *Gemmatimonadota* (linked to phosphorus solubilization) in T6 treatment (Figure 4). Future metagenomic and metabolomic analyses could further elucidate the metabolic pathways involved in these interactions. Previous studies have also found similar results. For example, Yue et al. (2023) and others found that microbial fertilizers can improve the soil fertility of saline-alkali land, improve soil physical and chemical properties, and thus affect the microbial community structure. Wang et al. (2024) and others found that the rhizosphere microbial community has an important contribution to plant growth and health and is affected by the type and application amount of fertilizers. The results of this study are consistent with these studies, further confirming the important role of bacterial fertilizer and soil amendment in improving the soil environment and promoting plant growth.

This study revealed the regulatory differences of different treatment groups on soil microbial functions through functional prediction analysis, providing functional evidence for analyzing the impact of treatment measures on the soil ecosystem. In terms of carbon metabolism, the ACADM (acyl-CoA dehydrogenase) function enriched in the T1 and T2 treatments strengthened the fatty acid β-oxidation metabolism, which aligns with the research of He et al. (2012). This study indicated that the activation of enzymes related to fatty acid metabolism accelerates the decomposition of lipid substances in soil organic matter, optimizes carbon source utilization efficiency, and promotes soil carbon cycling. The regulation of the frc (formyl-CoA transferase) function in the T5 treatment further demonstrates that treatment measures can reshape the branching flow of soil carbon metabolism by influencing the formic acid metabolism pathway, echoing the complexity of the microbial carbon metabolism network (Mooshammer et al., 2014). Regarding the correlation between nitrogen metabolism and soil fertility, the lysY (lysine metabolism-related enzyme) function significantly enriched in the T6 treatment directly participates in organic nitrogen decomposition, improving nitrogen availability. This is consistent with the conclusion proposed by Shu et al. (2024) that “microbial nitrogen metabolism functional genes are key drivers of the soil nitrogen cycle.” Additionally, the enhancement of the iucC (siderophore synthase) function in the T3 treatment not only promotes competitive iron absorption by microorganisms but also indirectly optimizes the soil-plant nutrient interaction process by improving plant iron nutrition absorption, which matches the research of M Craig et al. (2012) on siderophores regulating soil nutrient cycles. Differences in microbial stress resistance functions also merit attention. The enrichment of the gabD (succinic semialdehyde dehydrogenase) function in the T3 treatment enhances microbial stress resistance by strengthening γ-aminobutyric acid (GABA) metabolism, aligning with the view in Ramos-ruiz et al. (2019) that “the GABA metabolic pathway is an important mechanism for microorganisms to cope with environmental stress.” In this study, T6 treatment significantly enhanced the stress resistance (such as omp31) and nitrogen metabolism function (such as lysY) of microorganisms. This forms a synergistic effect with the stable maintenance of alkali-hydrolyzable nitrogen and Olsen-P in the soil (Figure 2) and the deep penetration of roots (Figure 1D). This functional optimization may ultimately support the yield increase of *Spuriopinella brachycarpa* (Kom.) Kitag. (Table 2) by promoting rhizosphere nutrient cycling and plant-microbe signal transduction. Among them, the optimization of nitrogen metabolism and stress resistance functions in the T6 treatment provides a mechanistic explanation—at the microbial function level—for its improvement of crop yield and soil fertility. It also confirms the research consensus that “microbial function regulation is an important pathway for soil ecological optimization” (Dong et al., 2024).

However, this study still has some limitations. First, although the soil microbial community was analyzed by sequencing technology, there was relatively little research on the function of the microbial community. Future research can combine metagenomics, transcriptomics, and other technologies to deeply explore the relationship between the function of the microbial community and the growth and development of *Spuriopinella brachycarpa* (Kom.) Kitag. and clarify the specific mechanism of microorganisms in soil nutrient cycling, plant hormone regulation, and other processes. Second, this experiment was only conducted for one growing season, and the long-term effect is still unclear. Long-term continuous application of bacterial fertilizer and soil amendment may have a cumulative impact on the soil ecosystem. Therefore, long-term positioning experiments are needed to evaluate the impact of these treatment measures on soil quality, microbial community stability, and the sustainable production of *Spuriopinella brachycarpa* (Kom.) Kitag. In addition, this study only considered the combination of two types of bacterial fertilizers and one soil amendment. Future research can further explore the effects of different types and proportions of bacterial fertilizers, soil amendments, and their combinations on the growth of *Spuriopinella brachycarpa* (Kom.) Kitag. and the soil environment and screen out more optimized treatment schemes to provide a more scientific basis for the efficient cultivation of *Spuriopinella brachycarpa* (Kom.) Kitag.

## 5 Conclusion

### 5.1 Growth and quality improvement

The combined application of Trichoderma harzianum and Bacillus subtilis fertilizers significantly increased yield by 30% and enhanced nutritional quality, particularly in T6 treatment.

### 5.2 Root system optimization

T6 treatment promoted root penetration depth by 50%, likely due to improved soil structure from earthworm polysaccharide.

### 5.3 Soil fertility maintenance

T6 maintained 127 mg/kg alkali-hydrolyzable nitrogen and 41 mg/kg Olsen-P at 60 days through microbial nutrient cycling.

### 5.4 Microbial community enhancement

T6 achieved the highest Chao1 index (3465) and Shannon diversity (10.84), enriching beneficial genera Nocardioides and Gemmatimonas.

### 5.5 Microbial function prediction

This study revealed novel functional enrichments in T6, including omp31 for stress resistance and lysY for nitrogen metabolism, providing mechanistic insights into yield improvement.

## Data Availability

The raw sequence data reported in this paper have been deposited in the Genome Sequence Archive in National Genomics Data Center, China National Center for Bioinformation/Beijing Institute of Genomics, Chinese Academy of Sciences (GSA: CRA024743) that are publicly accessible at https://ngdc.cncb.ac.cn/gsa.
